# Clutch Control: Changing the Speed and Direction of CAR-T Cell Therapy

**Published:** 2022

**Authors:** Alberto Nobili, Aya Kobayashi, Patrick C. Gedeon, Carl D. Novina

**Affiliations:** 1 Department of Cancer Immunology and Virology, Dana-Farber Cancer Institute, Boston, MA 02215, USA; 2 Department of Medicine, Harvard Medical School, Boston, MA 02115, USA; 3 Broad Institute of Harvard and MIT, Cambridge, MA 02142, USA; 4 Current Address: Dynamic Cell Therapies, Inc., 127 Western Ave., Allston, MA 02134, USA; 5 Department of Surgery, Brigham and Women’s Hospital, Boston, MA 02115, USA; 6 Department of Surgery, Harvard Medical School, Boston, MA 02115, USA

## Commentary

The adoptive transfer of T cells expressing chimeric antigen receptors (CAR-T cells) has revolutionized the treatment of hematological malignancies, providing unmatched clinical responses in adults and children with relapsed or refractory B cell malignancies. To date, this has led the US Food and Drug Administration (FDA) to approve six separate CAR-T cell therapies [1–6]. The success of CAR-T cell therapy for treating solid tumors, however, has been limited [7–11]. There are several possible reasons for this including intratumoral heterogeneity, lack of tumor-specific proteins universally expressed on the surface of tumor cells, and adverse consequences due to off-tumor and uncontrolled immune activity.

Indeed, less than half of the patients treated with CAR-T cells achieved long-term durable remission [4,12,13]. As of 2022, approximately 15,000 patients with hematological malignancies have been treated with CAR-T cells with an average of approximately 400 CAR-T cell infusions per month in the US alone, and response rates of 60-to-90% worldwide [14–16]. Despite widespread use, relapse has been shown to occur for many patients who initially respond; a phenomenon thought to be due, in part, to downregulation or loss of CAR-targeted protein expression on the surface of tumor cells [17,18]. Other obstacles to CAR-T cell therapy for solid tumors include poor persistence of CAR-T cells after infusion, insufficient CAR-T cell tumor infiltration, and T cell exhaustion [19], together resulting in inefficient or slow kinetics of tumor cell lysis, further increasing the chances of tumor evolution and escape from CAR-T cell therapy.

To overcome these limitations, we recently described a novel binary-activated CAR-T cell capable of simultaneously targeting multiple tumor antigens in a precise and controllable fashion [20]. By engineering the CAR-T cell to recognize a small molecule and then conjugating the same small molecule to distinct tumor-targeting antibodies, a single small-molecule-specific CAR-T cell can be directed against multiple separate targets to increase the kinetics of tumor-cell lysis. To enable safe targeting of cell surface tumor antigens shared with non-neoplastic cells, we incorporated an ultraviolet (UV) light-sensitive cage coupled to small molecules that inhibits CAR-T cells from interacting with the small molecules until exposure to UV light. This caging strategy can be used to increase the therapeutic window of CAR-T cells, allowing for temporal and spatial control of CAR-T cell activity. We believe that such binary-activated CAR-T cells provide a dynamic and adaptable approach that will accelerate the kinetics of tumor cell lysis while increasing the therapeutic window, allowing for safe and effective treatment of solid tumors. In this Commentary, we review this and other novel therapeutic approaches and discuss their potential utility for treating solid tumors.

## Challenges

### Identifying safe and effective CAR-T cell target antigens

The majority of driver oncogenes are intracellular and are not targetable by conventional CAR-T cells [21]. Accordingly, all FDA-approved CAR-T cell therapies target abundantly expressed cell-surface proteins (e.g. CD19 and B-cell maturation antigen (BCMA)). Most of these transmembrane proteins are not related to oncogenesis, but instead are related to tumor cell lineage, with tumor cells originating from B cells or plasma cells, in the case of CD19- and BCMA-targeted CAR-T cells, respectively. Although non-neoplastic B cells are depleted by CD19-targeted CAR-T cell therapy, the depletion of B cells, and the resulting agammaglobulinemia is clinically manageable as elimination of B cells is compatible with life.

For treating solid tumors, however, target antigens are often also expressed on the surface of healthy cells, and even low-level expression of a target protein on the surface of healthy tissue can produce on-target but off-tumor toxicities. In some cases, CAR-T cell therapies have led to severe, and even fatal, toxicities due to the on-target but off-tumor activity directed against healthy tissues. For example, CAR-T cells directed against human epidermal growth factor 2 (HER2), a transmembrane tyrosine kinase in the ERBB family, has been used for the treatment of breast, gastric, and colorectal cancers as well as medulloblastoma [22–25]. Unfortunately, even low-level expression of HER2 on normal pulmonary epithelial cells led to pulmonary toxicity as well as cytokine release syndrome (CRS) [26–28]. CAR-T cells targeting carcinoembryonic antigen-related cell adhesion molecule 5 (CEACAM5) used for the treatment of breast, gastric, colorectal, lung, ovarian, and pancreatic cancers induced acute respiratory toxicity, likely due to CEACAM5 expression on non-neoplastic lung epithelium [29]. Similarly, metastatic renal cell carcinoma patients treated with CAR-T cells targeting carboxy-anhydrase-IX (CAIX) developed cholangitis due to the on-target but off-tumor toxicity such as the destruction of CAIX-expressing bile duct epithelial cells [30]. Indeed, most solid tumors do not express a unique tumor-specific surface protein that is absent from healthy tissues. There is a need for new technologies that allow for safely targeting tumor-associated, cell-surface proteins without associated on-target, off-tumor toxicities.

Others have shown that CAR-T cells directed against tumor-associated cell surface targets can be used safely, without dose-limiting toxicities, though efficacy has been limited. This has included CAR-T cells directed against epidermal growth factor receptor (EGFR) [31,32], interleukin 13 receptor subunit alpha 2 (IL13Ra2) [33], mesothelin [34–36], carcinoembryonic antigen [CEA] [37,38], prostate-specific membrane antigen (PSMA) [39], and disaloganglioside GD2 [40,41]. While these CAR-T cells have demonstrated reasonable safety profiles, clinical benefit has been limited. The safety of these targets for CAR-T cell therapy may need to be reevaluated when effective anti-tumor responses are achieved.

### Tumor heterogeneity

A major challenge to successfully implementing CAR-T cell therapy for patients with solid tumors is inter-tumoral and intra-tumoral heterogeneity. Targetable antigens can differ among patients with the same type and stage of cancer, limiting the utility of a single-antigen targeted CAR-T cell therapies. Furthermore, there is heterogeneity within individual patient’s tumors, with sub-populations of tumor cells expressing different levels of the same antigen, different isoforms of the same antigen or different antigens entirely. This is important as CAR-T cell-induced tumor cell lysis is directly proportional to the level of the target antigen expressed on the surface of tumor cells [42–46]. Tumor cells with reduced or absent expression of CAR-T cell-targeted antigens below the threshold required for CAR-T cell activation result in inefficient tumor cell lysis and are a potential source of treatment failure or recurrence, highlighting a limitation of single antigen-targeted CAR-T cell therapy.

Although some patients achieve long-term durable responses after treatment with single-antigen targeted CAR-T cell therapies [4,12,13], recurrent disease associated with downregulation or loss of target antigen expression on tumor cells is a significant challenge [17,18]. In patients with CD19-positive lymphomas, durable, complete remission rates of 30–50% have been achieved with lymphodepleting chemotherapy followed by adoptive transfer of CD19-targeted CAR-T cells [47,48]. However, disease relapse is associated with the emergence of antigen-negative tumor cells, resulting in treatment failure [49–51].

To combat intratumoral evolution and antigen escape known to occur with the relatively slower kinetics of single-antigen targeted CAR-T cell therapy, we and others have evaluated strategies using multi-antigen targeted T cells in both hematological [52,53] and solid tumors [43,54–57]. For example, Ciltacabtagene autoleucel is a CAR-T cell therapy expressing two BCMA-targeting single-domain antibodies that has been approved for treating patients with multiple myeloma [58], demonstrating the safety of a multi-epitope targeting approach. Others have alternatively infused separate populations of CAR-T cells, each of which target different antigens. This has included clinical evaluation of CAR-T cells directed against CD19 and CD22 for B-lymphoblastic leukemia [59], BCMA and CD19 for multiple myeloma [60], and EGFR and CD133 for cholangiocarcinoma [57]. Some groups have used bivalent CAR-T cells targeting two distinct antigens. These include CAR-T cells simultaneously targeting CD19 and CD20 [61,62], CD19 and CD22 [63], CD19 and CD123 [53], BCMA and SLAM family member 7 (SLAMF7) [64], BCMA and CD38 [65], and glypican 2 (GPC2) and CD276 (B7-H3) [66,67]. Trivalent CAR-T cells simultaneously targeting CD19, CD20 and CD22 [68] and HER2, ephrin type-A receptor 2 (EphA2) and IL13Ra2 [69] have also been explored. These bivalent and trivalent CAR-T cells achieved better clinical and preclinical responses in comparison to monovalent CAR-T cells, highlighting the utility of such a multi-antigen targeted CAR-T cell approach.

Together, these data suggest that targeting multiple antigens simultaneously is safe and leads to more effective responses when compared to targeting single antigens alone. Moreover, targeting multiple epitopes simultaneously likely leads to more rapid clearance of tumors, decreasing the likelihood of intratumoral evolution and antigen escape, thereby producing more durable responses. While these strategies demonstrate the utility and safety of combinatorial epitope targeting with CAR-T cell therapy, they are limited in that they require separate viral transduction of multiple CAR-T cell constructs or are limited in scope to the initial targets of the infused CAR-T cell population [43,52–69]. In contrast, our strategy utilizing small-molecule targeted CAR-T cells is adaptable, being capable of simultaneously targeting multiple tumor antigens following the infusion of a single CAR-T cell construct [20].

## Opportunities

### Switch-controls increase the therapeutic window for CAR-T cell therapies

One method to redirect CAR-T cells against multiple antigens is to uncouple antigen binding from CAR-T cell engagement with tumor cells. This can be accomplished by using “switch-controlled” CAR-T cells which consist of two-parts. The first part is a CAR-T cell directed against a peptide tag, such as the 5B9 tag-CAR (UniCAR) [70] or GCN4 peptide-CAR [71,72], or against a small molecule such as biotin [73] or fluorescein (FL) [20,71,74,75]. The second part utilizes a tumor-targeting antibody conjugated to a protein, peptide, or small molecule. Such two-part systems enable CAR-T cell-mediated killing only when the antibody-conjugate is administered. Unlike conventional CAR-T cells with fixed antigen specificity, switch CAR-T cells can be redirected to multiple targets on the surface of tumor cells by using antibodies with distinct specificities, each coupled to the same protein, peptide, or small molecule. This allows for the timing and strength of CAR-T cell cytotoxic activity to be controlled in a small molecule-dose-dependent manner [20].

Conventional CAR-T cells with fixed antigen specificity have a narrow therapeutic window when used for the treatment of solid tumors where targeted antigens are commonly also found on non-neoplastic tissue. This results in on-target but off-tumor dose-limiting toxicities, as induced lysis occurs when CAR-T cells encounter cells expressing the target antigen, regardless of whether the cell is a cancer cell or non-neoplastic cell [76]. In contrast, the switch CAR-T approach allows for the adoptive transfer of a single population of T cells that can be redirected against separate antigens through the administration of separate small molecule-conjugated monoclonal antibodies. By choosing a combination of monoclonal antibodies with distinct antigen specificities and different on-target, off-tumor activities, the dose of each antibody can be optimized to minimize on-target, off-tumor activity while producing synergistic anti-neoplastic activity. Together, this results in increased anti-tumor activity with potentially decreased on-target, off-tumor activity, widening the therapeutic window and increasing the safety and efficacy of CAR-T cells directed against solid tumors [20].

We recently described switch CAR-T cells directed against different small molecules, including fluorescein and 4-[(6-methylpyrazin-2-yl)oxy]benzoic acid (MPOB) [20]. We anticipate that simultaneously targeting multiple antigens will accelerate the kinetics of tumor cell lysis and reduce tumor evolution and antigen escape. Furthermore, if CAR T cell therapy results in target-antigen negative recurrence, persisting switch CAR-T cells can be re-directed against recurrent tumor cells by administering additional small-molecule conjugated monoclonal antibodies specific for additional targets on recurrent tumor cells [70,77–80].

When compared to other switch-controlled CAR-T cells, our small-molecule strategy adds an additional level of control over CAR-T cell activity by allowing for titration of the density of small molecules conjugated to monoclonal antibodies. Consistent with the observation that CAR-T cell activation is correlated with the density of surface antigens on target cells [81,82], we observed increasing CAR-T cell activity with increasing density of small molecules conjugated to monoclonal antibodies. Thus, titrating the density of small molecules conjugated to antibodies along with the dose of the antibody-small molecule conjugate administered allows for further widening the therapeutic window of CAR-T cells for treating patients with solid tumors.

Additionally, we directed our switch CAR-T cells against small molecules, such as fluorescein, rather than peptides or larger proteins as others have done because small molecules are poorly immunogenic and have previously been used safely in the clinic. Indeed, fluorescein has been used in angiography without severe side effects [83] or as a tracer prior to tumor resection [84]. In contrast, switch CAR-T cells directed against peptides and proteins may be limited in efficacy by host immunity directed against the peptide or protein molecule. Previously developed switch CAR-T cells directed against the Fc portion of FDA-approved antibodies have also led to fatal side effects due to non-specific T cell activation, suggesting that careful selection of adaptor protein or peptide tags is important to precisely control switch CAR-T cell activity [85]. These data suggest that careful consideration must be given to the adaptor and linker molecules for precise control over CAR-T cell activity [85].

We have shown that anti-fluorescein CAR-T cells (FL-CAR-T cells) can target multiple antigens including EGFR, HER2, and CD38 expressed on the surface of the breast cancer, ovarian cancer, Burkitt lymphoma, and multiple myeloma cells. FL-CAR-T cells induced dose-dependent cytotoxicity of tumor cells labeled with antibody-fluorescein conjugates; this tumor cell killing was proportional to both the concentration of antibody-fluorescein complexes and the density of fluorescein conjugated to a tumor-targeting antibody. These data suggest that our small molecule switch CAR-T cells may be a potent, safe, and effective CAR-T cell with “customizable kinetics” of target cell lysis that may be useful for treating patients with multiple types of cancer [20].

Furthermore, to model CAR-T cell-induced lysis of heterogenous populations of tumor cells, we mixed EGFR-expressing tumor cells with CD38-expressing tumor cells. Addition of fluorescein-conjugated anti-EGFR monoclonal antibody resulted in cell lysis of EGFR-expressing tumor cells while addition of fluorescein-conjugated anti-CD38 monoclonal antibody resulted in cell lysis of CD38-expressing tumor cells. The combination of both monoclonal antibodies, however, resulted in the killing of both EGFR and CD38 expressing tumor cells, demonstrating that single-specificity CAR-T cells can be used to simultaneously target multiple tumor antigens and kill heterogeneous populations of cells [20]. Such an approach could accelerate the kinetics of tumor cell lysis and prevent antigen escape while maintaining a high level of safety.

### Local control over access to CAR-T cell antigens, and future directions

While our switch CAR-T cell system allows for antigen multiplexing and titratable control over CAR-T cell activity, finding the right combination of antibodies with both meaningful on-tumor and minimal off-tumor activities can be challenging, especially in the context of treating patients with solid tumors. To overcome this limitation and further improve the safety of our switch CAR-T cell system, we implemented ultraviolet (UV) light-sensitive cage technology to our small molecule switch CAR-T cell system. Two near UV light-sensitive 5-carboxymethoxy-2-nitrobenzyl (CMNB) caging groups covalently coupled to the CAR-T cell-targeted small molecule prevented recognition of small molecules by CAR-T cells. This CMNB-cage is stable under the light of an operating room or in physiologic environments but is destabilized by exposure to the UV-light at 365nm, leading to the release of the CMNB-cage [20]. Once uncaged, the anti-small molecule CAR-T cell can recognize the small molecule and engage in tumor cell lysis. This strategy is particularly relevant in the context of solid tumors where small molecule-coupled antibodies bound to the surface of the tumor cells can be selectively exposed to UV light. Exposing the small molecule to UV light results in uncaging, CAR-T cell recognition, and subsequent tumor cell lysis, while small molecule-coupled antibodies bound to targets on distant, non-neoplastic cells remain caged and thus protected from CAR-T cell attack. This caging strategy enables temporal and spatial control over tumor cell killing while reducing the risk of on-target, off-tumor toxicities. The caging strategy can be alternated with dosing uncaged small molecule-coupled antibodies or mixed with small molecule-coupled antibodies directed against antigens with a more tumor-specific expression pattern, thereby generating a wider therapeutic window overall [20].

While this caging strategy may be useful for targeting tumor antigens broadly expressed on healthy tissue, it may also be implemented to safely enhance CAR-T cell tumor penetration. Stromal cells in the tumor microenvironment secrete extracellular matrix and create a physical barrier that prevents the infiltration of CAR-T cells and other immune cells into tumors [86]. CAR-T cells have been developed to attack fibroblasts in the tumor microenvironment, resulting in increased stromal permeability and improved immune cell infiltration into solid tumors [86]. However, most proteins expressed by tumor stroma are also widely expressed in non-neoplastic tissue, the targeting of which can lead to life-threatening on-target, off-tumor toxicities, especially secondary to widespread destruction of the bone marrow [86]. An intermittent CAR-T cell activity, like the one provided by the use of the caged small molecule and switch CAR-T cell platform, however, may allow for precise targeting of intratumoral stromal cells without associated wide-spread tissue destruction [87].

One limitation of translating this UV-caging strategy to clinical practice is insufficient tissue penetration by UV-light to achieve significant uncaging of small molecules within deeper tissues [88]. However, several photocaging groups are destabilized within the optical window for photomedicine applications, between 650 to 1300 nm [89], such as BODIPY or cyanine, and can be uncaged at longer (near infrared) wavelengths [90,91]. As such, our caged small molecule-targeted CAR-T cell system can be integrated with novel photocaging technologies and clinical applications as they are developed.

Other caging strategies that circumvent the need for light take advantage of secondary signals that are specific to the tumor microenvironment, such as tumor-specific proteases or the release of reactive oxygen or nitrogen species (ROS and RNS) [92–94]. The Probody approach developed by CytomX [95] uses an antibody coupled to a peptide that masks the antigen recognition site. The mask is connected via a protease-cleavable linker that prevents the binding of the antibody to the antigen. The Probody can be unmasked by proteases commonly found in the tumor microenvironment. Combining antibody masking and small molecule caging strategies could increase tumor-specificity and expand the number of targetable antigens. These modular approaches will increase the specificity and efficacy of CAR-T cell therapies and reduce the risk of toxicity and relapse for heterogenous solid tumors.

Our caged small molecule CAR-T cell system may be complemented by bio-orthogonal technologies. For example, fluorescein- or metal-labelled antibodies could be used to track initial tumor heterogeneity and tumor evolution in response to therapy [96–98]. Image segmentation could be used to (i) predict the density and distribution of target proteins in tumors (innate and acquired tumor heterogeneity), (ii) mark the boundaries of the targeted region, and (iii) provide visual confirmation of which target(s) were effective in the therapy of heterogeneous tumors [99–102].

While CAR-T cell therapy is approved for relapsed, refractory disease, recent clinical trials have suggested that CD19-directed CAR-T cell therapy may be more effective than the standard of care second-line therapy for patients with non-Hodgkin lymphoma [103–105]. These results support the use of CAR-T cell therapy for earlier stage disease. As this occurs, it will be important to adopt novel CAR-T cell strategies that enhance the safety profile by widening the therapeutic window while allowing for increased diversity of targets.
